# Giant frequency down-conversion of the dancing acoustic bubble

**DOI:** 10.1038/srep37385

**Published:** 2016-11-18

**Authors:** P. A. Deymier, M. Keswani, N. Jenkins, C. Tang, K. Runge

**Affiliations:** 1 Department of materials Science and Engineering, 1235 E. James E. Rogers Way, University of Arizona, Tucson AZ 85721, USA.

## Abstract

We have demonstrated experimentally the existence of a giant frequency down-conversion of the translational oscillatory motion of individual submillimeter acoustic bubbles in water in the presence of a high frequency (500 kHz) ultrasonic standing wave. The frequency of the translational oscillations (~170 Hz) is more than three orders of magnitude smaller than that of the driving acoustic wave. We elucidate the mechanism of this very slow oscillation with an analytical model leading to an equation of translational motion of a bubble taking the form of Mathieu’s equation. This equation illuminates the origin of the giant down conversion in frequency as arising from an unstable equilibrium. We also show that bubbles that form chains along the direction of the acoustic standing wave due to radiation interaction forces exhibit also translation oscillations that form a spectral band. This band extends approximately from 130 Hz up to nearly 370 Hz, a frequency range that is still at least three orders of magnitude lower than the frequency of the driving acoustic wave.

The complex behavior of bubbles in acoustic fields has fascinated generations of professional and amateur scientists. Acoustic bubbles can produce sonochemical reactions^1^, can emit light^2^,^3^, are used in therapeutic applications such as drug delivery^4^, or can be used to activate genetically targeted neurons^5^. The chemical and physical behavior of acoustic bubbles is associated with a wide spectrum of phenomena^6^,^7^ ranging from stable and unstable radial oscillations, rectified diffusion, microstreaming and translational motion including erratic “bubble dancing”^8^,^9^,^10^,^11^. Eller and Crum^12^ explained erratic bubble dancing via surface instability of the bubble. Numerical simulations have suggested the possible existence of slow oscillatory translational motions of bubbles in standing waves^13^,^14^,^15^,^16^. The frequency of these translational oscillations is anticipated to be several orders of magnitude lower than the frequency of the acoustic wave that drives the bubble motion. Here we show experimentally the existence of these very low frequency translational oscillations of bubbles in an acoustic standing wave. We elucidate the mechanism of the slow rhythmic dance of acoustic bubbles with the simplest analytical model and illuminate the origin of the giant down-conversion in frequency of this periodic motion. The mechanism described here which couples the fast radial motion and a slow translational motion of bubbles in an acoustic field suggests the capacity to shape bubble trajectories. Directing bubble trajectories through the control of acoustic fields may have important implications in the area of guided transport of chemical, biochemical or other agents, when considering bubbles loaded with gaseous cargoes or liquid droplets^17^.

We investigate experimentally, the translational motion of submillimeter-size bubbles in deionized (DI) water saturated with air at pH 5.8 subjected to an acoustic standing wave field of 500 kHz. The bubbles formed during sonication are therefore air bubbles. The typical diameter of bubbles characterized for translation motion is ~0.35 mm. These bubbles are about 25-fold larger than the resonant bubbles (diameter 0.015 mm) at 500 kHz excitation frequency, which ensures that the bubbles exhibit only small amplitude non-resonant radial oscillations at the driving frequency. The submillimeter-size bubbles localize at the antinodes of the standing wave due to first-order Bjerkness forces^6^,^7^. Bubbles will also form stable chains due to secondary Bjerkness forces^18^ resulting from bubble-to-bubble interactions via the secondary sound fields produced by their radial oscillation. Using a high resolution high speed video camera we record the translational motion in the direction of the acoustic wave, *x*(*t*), of individual bubbles as well as of bubbles in chains (See Fig. 1).

Figure 2 illustrates the typical translational behavior of a single bubble. The bubble position, *x*(*t*), normalized to half the standing wave wavelength, *λ*/2, exhibits what appears to be oscillatory behavior. To reduce the effect of possible random displacements of the bubble, we calculate the normalized position time-autocorrelation function which when fast Fourier transformed produces the bubble vibrational power spectrum. The most significant feature of the power spectrum of the single bubble is the existence of a well-defined peak at approximately 170 Hz. The very low frequency feature in the spectrum is assigned to the erratic behavior of the bubble. Indeed, we have verified that numerically generated random walks constrained by the experimental velocity of the bubble and the experimentally observed amplitude of the oscillation generate only power spectra features with frequency lower than 100 Hz. Five other submillimeter-size single bubbles that were studied showed qualitatively the same behavior and semi-quantitatively the same features in their power spectrum. The observed peak at 170 Hz is therefore characteristic of a translation oscillatory motion of the bubble trapped and driven by the standing wave. Variability in the position of the translational oscillation peak is observed with frequency ranging between 150–200 Hz. The frequency of this oscillatory motion is nearly 3000 times lower than the frequency of the driving acoustic wave. It was shown theoretically that bubble levitation during single-bubble sonoluminescence (SBSL) conditions is accompanied by translational oscillations^19^. However, the translational motion of the 4 μm bubble varies almost sinusoidally with the same frequency as the acoustic standing wave (25 kHz). These translational oscillations are intimately coupled with the highly non-linear radial oscillations of the sonoluminescence bubble during its periodic collapse. This condition is quite different from that of our sub-millimeter bubbles that exhibit small amplitude radial oscillations. Experimental observation of frequency down-converted translational oscillations of an acoustically levitated micron-size bubble in aqueous ethanol solutions has also been reported^20^. The translational oscillations were shown to depend drastically on the concentration in ethanol. There was no translational motion in pure water even when the bubble was driven at the maximum acoustic pressure. In presence of ethanol, the translational oscillations have periods on the order of seconds for an acoustic driving frequency of 26 kHz. As the radius oscillates at the driving frequency, the period of the translational motion is related to the variation in the maximum bubble radius resulting from the ethanol reaction and diffusion processes occurring at the gas/liquid interface.

The conditions of our experiments are far from SBSL conditions. Based on our high resolution imaging, we note that the radial dimension of the submillimeter bubble in our system appears not to vary during the translational oscillations. The bubbles do oscillate radially as shown indirectly by the presence of chains of bubbles. Indeed, chains of bubbles will form due to secondary Bjerknes bubble-to-bubble forces resulting from secondary sound fields emitted by the bubbles radial oscillations. However, the amplitude of these oscillations for sub-millimeter bubbles is small in contrast to that observed in SBL experiments. The frequency of the radial oscillations of sub-millimeter bubbles is also expected to be that of the driving acoustic field. Because of their high frequency and small amplitude, these radial oscillations are therefore not directly observable with the high speed camera we used (see Experimental Method section). In contrast to SBSL experiments, the submillimeter bubbles of our experiments undergo radial oscillations with very small amplitude compared to the amplitude of a sonoluminescence bubble during its periodic collapse. Therefore, coupling between the translational motion and weak radial oscillations cannot explain the observed frequency-down converted translational oscillations. Furthermore, all our experiments are conducted in pure water and the giant frequency down-conversion in the translational motion of the bubble that we observe cannot be attributed to mass transport and/or reaction processes as is the case in ref. 20. To our knowledge, the remarkable giant frequency down-conversion we observe has not been reported before.

In Fig. 3, we report the typical translational behavior of a bubble in a chain. The bubbles are located at anti-nodes of the standing wave and separated by half a wavelength. The normalized bubble position, 2*x*(*t*)/*λ*, exhibits a well-defined oscillatory behavior which is also clearly apparent in the time-autocorrelation function. In contrast to the isolated bubble, the vibrational power spectrum of a bubble in a chain now exhibits translational oscillatory motion with a range of frequencies. This range extends approximately from 130 Hz up to nearly 370 Hz. Other submillimeter-size bubbles in chains that were studied showed qualitatively a similar behavior and a quantitatively similar frequency range. This range is characteristic of a band of translation oscillatory modes that results from the periodicity of the chain and of the interactions between bubbles and their associated secondary acoustic fields. The frequency of this band is still at least three orders of magnitude lower than the frequency of the acoustic wave. This behavior was verified for two bubbles in two different chains.

We show in the supplementary material that the translational dynamics of models of submillimeter single bubbles and bubbles in a linear chain located at the antinodes of an acoustic standing wave of frequency *ω* in a fluid can be described by an equation that takes the form of Mathieu’s equation^21^: 
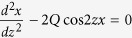
, where 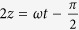. For the single bubble, we have 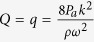 where *ρ* is the density of the fluid and 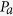 is the pressure amplitude of the acoustic wave with a wave number 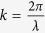 (*λ* being the wavelength). For a bubble in a chain 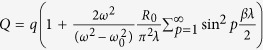, where *R*_0_ is the average radius of the bubble and 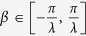 is a wave number characterizing the dispersion of the translational oscillations in the chain. The summation accounts for the long range nature of the interaction of bubbles and *p* refers to the order of interaction with neighboring bubbles (*p* = 1 for first-nearest neighbor interactions, *p* = 2 for second-nearest neighbor interactions, etc.). We have assumed that the radial oscillations of bubbles in a chain are in phase. This assumption results from our experimental observation of the formation of chains of bubbles which indicates that the radiation force between bubbles is attractive. It is well known that attractive radiation forces between two bubbles of similar size arise when their radial oscillations are in phase^6^. Further motivation for the fact that the bubbles are in phase is that they are arranged periodically in the standing wave field. For random arrays of bubbles one would not expect in phase oscillations of the bubbles except for special circumstances^22^. The coefficient 2*Q* cos 2*z* in Mathieu’s equation is a trigonometric periodic function that drives the translational dynamics of the bubble. The realm of application of Mathieu’s equation is quite broad. The prototypical examples of mechanical systems that obey Mathieu’s equation are the inverted pendulum^23^ as well as the floating vessel on a wavy sea^24^. Mathieu’s equation also describes the behavior of neutral and charged particles in electro-magnetic traps such as the 1989 Nobel-prize winning “Paul trap”^25^. The small-amplitude behavior of the surface oscillation of pulsating bubbles in an acoustic field has also been shown to obey a Mathieu relation^6^. For many of these systems, the primary focus has been on periodic solutions of the equation with the same period (*π*) or twice the period (2*π*) of the driving trigonometric function. To support the experimental observation of giant frequency down-conversion of translational oscillation motion of bubbles, the present study focuses on solutions that have not been appreciated in the literature, namely vibrational modes with frequencies that are significantly lower than that of the driving trigonometric function. While *x*(*t*) = 0 is a stable solution, we show, in the supplementary material, the existence of very low frequency solutions. These low frequency translational oscillations are representative of unstable dynamical equilibria. For the single bubble, the lowest frequency mode solution of Mathieu’s equation is found to be: 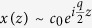. The onset of the low frequency oscillations requires that there be an initial translation velocity for the bubble and the amplitude, but not the frequency, of these oscillations depends on the bubble’s initial velocity. For water ρ = 1000 kg/m^3^, the longitudinal speed of sound is *v* *~* 1500 m/s, and with *ω* = 500 *kHz*, we determine *q* ~ 3.55 × 10^−9^ *P*_*a*_. We have measured in our experimental set up a pressure amplitude of 2.3 atm leading to *q* ~ 8.16 × 10^−4^ and a frequency for the translational oscillation of 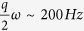. This value is in very good agreement with the experimental observations of a giant frequency down-conversion. The translational motion of a bubble in a chain is also shown to be significantly down-converted but the additional feature of dispersion occurs for these bubbles. That is the frequency of the down-converted modes depends on a wavelength resulting from the periodicity of the chain *i.e*. the periodicity of the standing wave. The long-range acoustic interaction forces between bubbles that act as secondary sources of sound are at the origin of the broadening of the frequency band and dispersion arises from the lifting of the degeneracy of the oscillatory mode of non-interacting bubbles. In the case of a finite chain of bubbles, the band is replaced by a series of discrete non-degenerate modes whose frequency range depends on the number of bubbles in the chain *i.e*., the range of interaction between bubbles. Unlike the well-known elastic harmonic chain composed of masses and springs, the band supported by a chain of bubbles possesses a lower bound for the frequency of the long wavelength modes. Bubbles in a chain do not behave like point masses interacting via linear forces but behave like particles interacting nonlinearly through their secondary sound fields.

We have demonstrated experimentally the existence of a giant frequency down-conversion of the translational oscillatory motion of submillimeter bubbles in water in the presence of a high frequency ultrasonic standing wave. The frequency of the translational oscillations is more than three orders of magnitude less than that of the driving acoustic wave. This surprising phenomenon, which requires an initial bubble velocity, can be understood by placing oneself in the bubble inertial reference frame. Recalling that a standing wave is composed of two waves traveling in opposite directions, relative to its reference frame, a moving bubble experiences frequency up-shifted and frequency down-shifted acoustic waves. In the case of a single bubble, the acoustic radiation forces (Bjerkness forces) resulting from the two frequency shifted waves and acting on the bubble do not balance each other and lead to an effective acceleration. The time scale of this acceleration is much longer than that of the driving acoustic wave, thus leading to a low frequency rhythmic dance of the acoustic bubble.

## Experimental Method

**Figure 4:**
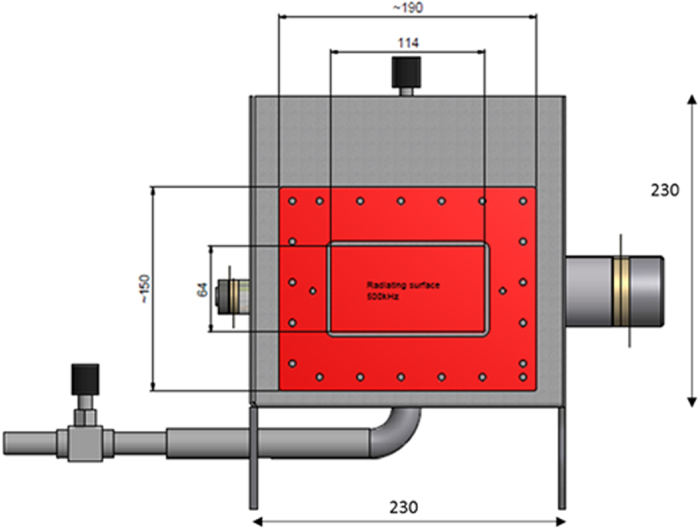
Schematic representation of the tank with transducer. All dimensions are in mm.

A custom designed rectangular stainless steel sonochemical reactor affixed with 500 kHz transducer on one of the side walls was used for the experimental work. The size of the sonochemical tank was 23 × 23 × 23 cm with transducer area of 73 cm^2^. The schematic of the tank with transducer is provided in Fig. 4. The reactor was filled with air saturated deionized (DI) water (8 liters) at 25 °C and a standing wave was formed when the sound wave reflected from the opposite side wall which was 23 cm away from the transducer surface. The maximum pressure amplitude of the wave was measured to be 2.3 atm using a HCT-0310 hydrophone (Onda Corporation, Sunnyvale, CA, USA) and a USB 5133 oscilloscope (National Instruments Corp., Austin, TX, USA) with acquisition rate set at 6 million samples per second. LabVIEW 2012 (version 12.0) was used to acquire the data, which was processed using Matlab 7.3. The set-up was equipped with a one megapixel digital high-speed camera (Phantom v711, Vision Research Inc., Wayne, NJ, USA) for imaging bubble trajectories at 4,000–10,000 frames-per-second (fps) and 1280 × 800 or 400 × 800 display resolution.

## Additional Information

**How to cite this article**: Deymier, P. A. *et al*. Giant frequency down-conversion of the dancing acoustic bubble. *Sci. Rep*. **6**, 37385; doi: 10.1038/srep37385 (2016).

**Publisher's note**: Springer Nature remains neutral with regard to jurisdictional claims in published maps and institutional affiliations.

## Supplementary Material

Supplementary Information

Supplementary Video S1

## Figures and Tables

**Figure 1:**
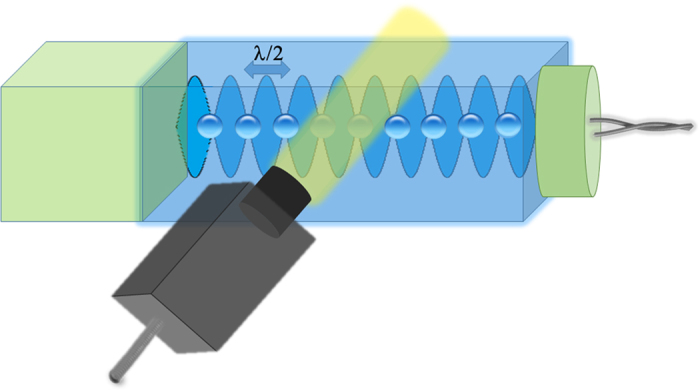
Schematic representation of a chain of bubbles in water supporting an acoustic standing wave of frequency. The standing wave is generated by reflection of an acoustic wave generated by a 500 kHz transducer (right). A high-resolution high-speed camera records the translational motion of bubbles with a sample rate ranging from 4,000 to 10,000 fps.

**Figure 2:**
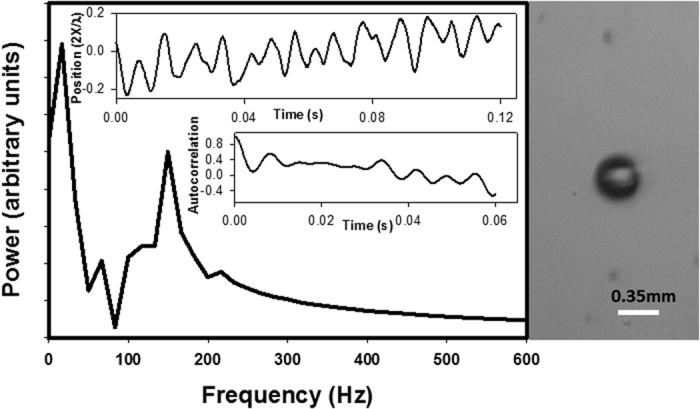
Oscillation power spectrum of a single bubble calculated as the Fourier transform of the normalized time-autocorrelation function of the position of the bubble (lower inset). Top inset: position of the center of the bubble normalized to half a wavelength of the acoustic standing wave as a function of time. Right: snapshot of the oscillating bubble.

**Figure 3:**
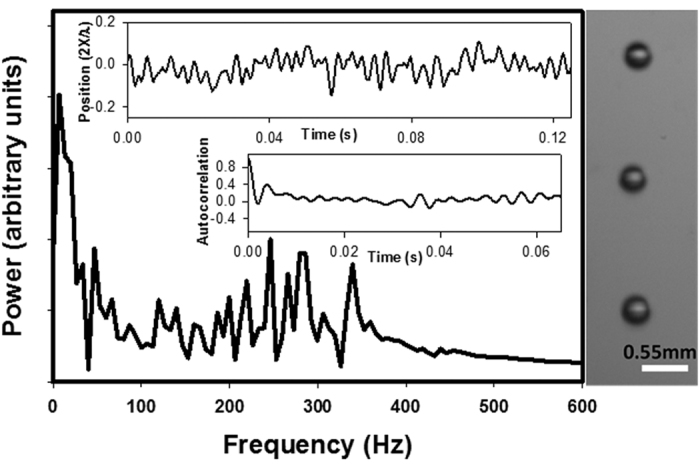
Oscillation power spectrum of a bubble in a chain of bubbles calculated as the Fourier transform of the normalized time-autocorrelation function of the position of the bubble (lower inset). Top inset: position of the center of the bubble normalized to half a wavelength of the acoustic standing wave as a function of time. Right: snapshot of a segment of a chain of bubbles. Data is reported for the central bubble.
