# Supplementary material for: Giant frequency down-conversion of the dancing acoustic bubble
Source: Sci Rep. 2016 Nov 18;6:37385. doi: 10.1038/srep37385 (PMC5114654; doi:10.1038/srep37385)
Supplement: Supplementary Information [file srep37385-s1.pdf]

Supplementary material

Giant frequency down-conversion of the dancing acoustic bubble

P.A. Deymier, M. Keswani, N. Jenkins, C. Tang and K. Runge

Department of Materials Science and Engineering

University of Arizona

Tucson AZ 85721

Video Legend:

Video of the dynamics of bubbles in a chain in a 500kHz standing wave in water. Time is shown at lower left corner of screen. The bubbles do not undergo radial oscillations as their diameter does not vary in time and remains at approximately 3/8 mm. The bubbles undergo only translational oscillatory motion.

Theoretical Models and Methods

1. Single bubble

Bubbles in an acoustic field are subjected to an effective acoustic radiation force

$$F(\boldsymbol{r},t) = -V(t)\nabla P(\boldsymbol{r},t) \quad (1)$$

where $V(t)$ is the time-dependent volume of the bubble and $\nabla P(r,t)$ is the local instantaneous pressure gradient. Under the influence of this effective force, a bubble may undergo oscillatory translations in response to an oscillatory pressure field associated with the sound wave as well as its own volume pulsation. These instantaneous oscillatory motion is expected to occur at the frequency of the acoustic field about a position that drifts slowly. The net translational drift of the bubble results from the fact that the time average of the acoustic radiation force, the so-called Bjerknes force, is nonzero. In absence of any other forces such as drag, the dynamics of the bubble can be simply described by the equation of motion:

$$m_A\ddot{\boldsymbol{r}} = F(\boldsymbol{r},t) \quad (2)$$

where $m_A = \rho V_A$ is the apparent mass of the bubble with ρ being the density of the fluid (a constant for an incompressible fluid) and $V_A = \frac{1}{2}V(t)$ being the apparent volume. Inserting equation (1) into (2), one notes that the translational motion of the bubble becomes independent of the volume of the bubble, namely

$$\ddot{\boldsymbol{r}} = -\frac{2}{\rho}\nabla P(\boldsymbol{r},t) \quad (3)$$

We consider a one-dimensional acoustic standing wave field with frequency ω and wave number $k = \frac{2\pi}{\lambda}$ (λ being the wavelength):

$$P(x,t) = 2P_a \sin \omega t \sin kd \tag{4}$$

In this equation, P_a is the pressure amplitude of the acoustic wave. Assuming that the bubble is located initially near an antinode $d = \frac{2n+1}{4}\lambda + x$ of the standing wave, equation (3) can be simplified to describe the translational motion, $x(t)$ of the bubble in one-dimension along the direction of the wave field:

$$\ddot{x} = -\frac{2}{\rho}\frac{\partial P(x,t)}{\partial x} = \frac{4P_a}{\rho} k \sin \omega t \sin kx \tag{5}$$

In the limit of small kx (i.e. long acoustic wavelength and small displacements), equation (5) can be linearized:

$$\ddot{x} - \frac{4P_a}{\rho} k^2 \sin \omega t \, x = 0 \tag{6}$$

Defining the variable z by $2z = \omega t - \frac{\pi}{2}$, i.e. redefining the origin of time or introducing a phase which does not impact the search for the Eigen values, equation (6) can be rewritten into the conventional Mathieu's equation:

$$\frac{d^2x}{dz^2} - 2q \cos 2z \, x = 0 \tag{7}$$

with $q = \frac{8P_a k^2}{\rho\omega^2} = \frac{8P_a}{\rho v^2}$. In defining the parameter q with have used the dispersion relation for the acoustic wave in the long wavelength limit: $\omega = vk$. The parameter q depends only on the physical characteristics of the fluid and the pressure amplitude of the acoustic wave. Equation (7) is a simplified form of Mathieu's equation. The coefficient $2q \cos 2z$ is a periodic function of time and Floquet's theorem can be used to seek solutions in the form:

$$x(z) = e^{i\gamma z} f(z) \tag{8}$$

where $f(z)$ is a periodic function of period π and γ is the characteristic exponent. Expanding the periodic function f in a Fourier series:$f(z) = \sum_{K=-\infty}^{K=+\infty} c_k e^{i2Kz}$ and inserting the sought solution (8) into equation (7) leads to an Eigen value problem with the recurrence relation:

$$c_K(2K+\gamma)^2 + q(c_{K+1} + c_{K-1}) = 0 \tag{9}$$

This Eigen value problem can be written in matrix form:

$$Hc = 0 \tag{10}$$

by defining the tridiagonal operator *H*:

$$\begin{pmatrix} 0 & q & (-4+\gamma)^2 & q & 0 & . & . & . \\ . & 0 & q & (-2+\gamma)^2 & q & 0 & . & . \\ . & . & 0 & q & \gamma^2 & q & 0 & . \\ . & . & . & 0 & q & (2+\gamma)^2 & q & 0 \\ . & . & . & . & 0 & q & (4+\gamma)^2 & q \end{pmatrix} \tag{11}$$

and the vector

$$c = \begin{pmatrix} . \\ . \\ c_{-2} \\ c_{-1} \\ c_0 \\ c_1 \\ c_2 \\ . \\ . \end{pmatrix} \tag{12}$$

The Eigen value problem produces nontrivial solutions when the determinant of the matrix H is equal to zero: $detH = 0$. The H matrix is infinite so one can approximate the problem by employing a truncated version of H to some order n: $detH_n = 0$. For instance, to second order (n=2), we can write $detH_2 = \begin{vmatrix} \gamma^2 & q \\ q & (2+\gamma)^2 \end{vmatrix} = 0$. This leads to the following approximate solutions for the characteristic exponent: $\gamma_1 = -1 \pm \sqrt{1+q}$ and $\gamma_2 = -1 \pm \sqrt{1-q}$. We consider the case of small q. Indeed for water using ρ=1000kg/m^3 and v~1500m/s, $q{\sim}3.5510^{-9}P_a$. We have measured in our experimental set up a pressure amplitude of 2.3 atm leads to q~8.16x10^{-4}. Therefore, when q is small, the possible values of the characteristic exponents are real:

$$\gamma = \begin{cases} \pm\frac{q}{2} \\ -2 \pm \frac{q}{2} \end{cases} \tag{13}$$

A real characteristic exponents imparts an additional periodic character to the motion of the bubble $x(t)$ through the prefactor $e^{i\gamma z}$ in equation (8). Of particular interest here, is the case of $\gamma = \pm\frac{q}{2}$. Let us take $\gamma = +\frac{q}{2}$, the bubble displacement truncated to second order now reads:

$$x(z){\sim}c_0 e^{i\frac{q}{2}z} + c_1 e^{i\left(2+\frac{q}{2}\right)z} \tag{14}$$

Since q is small, the second term of equation (14) corresponds to high frequency oscillations with a frequency that differs only slightly from that of the acoustic standing wave. Solving for the truncated Eigen vector (c_0, c_1) for small q, through equation (10) leads to $c_1 = -\frac{q}{4}c_0$ with c_0 is indeterminate. This indicates that the low frequency oscillations are representative of an unstable dynamical equilibrium. Indeed, consider equation (7), x=0, is a stable solution. The onset of the low frequency oscillations and the amplitude of these oscillations depend on the initial conditions of the velocity of the bubble.

2. Bubble in a chain of interacting bubbles

For chains of bubbles, secondary Bjerkness forces bind the bubbles (this is why the chain forms in the first place) so the slow periodic motion of the bubbles are now coupled leading to the formation of a frequency band. The pressure radiated by a radially oscillating bubble, "b", at a radial distance, *r*, is given by:

$$P_b = \rho \frac{R}{r}\left(R\ddot{R} + 2\dot{R}^2\right) \tag{15}$$

We consider the limit of small radial oscillation amplitudes, $R(t) = R_0(1 + \varepsilon(t))$ where R_0 is the radius at equilibrium. Considering the dynamics of the bubble radius in the regime of the linearized form of Rayleigh-Plesset equation, $\varepsilon(t)$ is solution of the equation:

$$\ddot{\varepsilon} + \omega_0^2 \varepsilon = -\frac{1}{\rho R_0^2} P(x,t) \tag{16}$$

In that equation, the pressure is given by equation (4) and the characteristic frequency of the bubble is defined through the relation: $\omega_0^2 = \frac{1}{\rho R_0^2}\left[3\gamma_0\left(P_0 + \frac{2\sigma}{R_0}\right) - \frac{2\sigma}{R_0}\right]$, with γ_0, σ, and P_0 standing for the ratio of the specific heat of the gas at constant pressure to that at constant volume, the gas-water surface tension, and the static pressure. For a bubble at the antinode of a standing wave and in the limit of very small displacements, we take $\sin kd \sim 1$ and find: $\varepsilon(t) = \frac{2P_A}{\rho R_0^2}\frac{1}{\omega^2 - \omega_0^2}\sin \omega t$. Using the linearized *R*(*t*) and its derivatives, discarding the second term in equation (15), we approximate the radiated pressure by:

$$P_b \sim -2P_a \frac{\omega^2}{\omega^2 - \omega_0^2}\frac{R_0}{r}\sin \omega t \tag{17}$$

In a chain of bubbles, we assume that a bubble "*n*" is subjected only to the radiation pressure of its nearest neighbors, "*n*-1" and "*n*+1". The equation of motion of the bubble "*n*" takes the form:

$$\ddot{x}_n = -\frac{2}{\rho}\left(\frac{\partial P(x_n,t)}{\partial x_n} + \frac{\partial P_{b,n+1}\left(r(x_{n+1},x_n),t\right)}{\partial x_n} + \frac{\partial P_{b,n-1}\left(r(x_n,x_{n-1}),t\right)}{\partial x_n}\right) \tag{18}$$

In equation (18), we have defined the radial distances $r(x_{n+1}, x_n) = \frac{\lambda}{2} + (x_{n+1} - x_n)$ and $r(x_n, x_{n-1}) = \frac{\lambda}{2} + (x_n - x_{n-1})$ where $\lambda/2$ is the equilibrium distance between bubbles in the chain (antinode to antinode distance of the standing wave). We have also assumed that the coupling between bubbles in the chain does not affect their Eigen frequency and that all bubbles oscillate in phase. Equation (18) becomes:

$$\ddot{x}_n + \frac{4P_a}{\rho}\frac{2R_0}{(\lambda/2)^3}\frac{\omega^2}{\omega^2 - \omega_0^2}\sin \omega t\,(x_{n+1} - 2x_n + x_{n-1}) - \frac{4P_a}{\rho}k^2 \sin \omega t\, x_n = 0 \tag{19}$$

In deriving Eq. (19), we have used the approximation: $\frac{1}{\left[1+\frac{x_{n+1}-x_n}{\lambda}\right]^2} \sim 1 - 2\frac{x_{n+1}-x_n}{\lambda}$. The second term in equation (19) introduces spectral dispersion. This is best seen if we seek solutions in the form:

$$x_n(t) = x(\kappa, t)e^{i\beta n\lambda} \tag{20}$$

with $\beta \epsilon \left[-\frac{\pi}{\lambda}, \frac{\pi}{\lambda}\right]$. With this, equation (19) reduces to:

$$\ddot{x}(\beta, t) - \frac{4P_a}{\rho} k^2 \left(1 + \frac{2\omega^2}{(\omega^2-\omega_0^2)} \frac{8R_0}{\pi^2\lambda} \sin^2 \frac{\beta\lambda}{2}\right) \sin \omega t \, x(\beta, t) = 0 \qquad (21)$$

Again, introducing the variable z, the equation of motion (21) takes the same form as equation (7):

$$\frac{d^2x(\beta,z)}{dz^2} - 2q' \cos 2z \, x(\beta z) = 0 \qquad (22)$$

with $q'(\beta) = q\left(1 + \frac{2\omega^2}{(\omega^2-\omega_0^2)} \frac{8R_0}{\pi^2\lambda} \sin^2 \frac{\beta\lambda}{2}\right)$. The slow oscillatory translational motion of bubbles in the chain is dispersive with frequencies given by: $\gamma(\beta)\omega = \frac{q'(\beta)}{2}\omega$. The translational oscillations form a band in the interval: $\frac{q}{2}\omega\left[1, 1 + \frac{2\omega^2}{(\omega^2-\omega_0^2)} \frac{8R_0}{\pi^2\lambda}\right]$. For millimeter and submillimeter size bubbles, $\omega^2 \gg \omega_0^2$, this simplified model predicts a bandwidth significantly smaller than the one observed experimentally due to the linear approximation for the radial and translational dynamics as well as the restriction to first nearest neighbor assumption for the bubble-bubble interaction. However, since the bubble-to-bubble separation distance in a chain is on the order of the wavelength of the secondary sound waves emitted by the radially oscillating bubbles, other secondary Bjerknes forces beyond the first-nearest neighbor interaction should also be accounted for. In water, one does not expect damping of the secondary Bjerknes forces beyond distance amounting to many wavelengths i.e. many inter-bubble distances. If we account for interactions to second-nearest, third-nearest, and higher order nearest neighbors, then equation (19) reads:

$$\ddot{x}_n + \frac{4P_a}{\rho} \frac{2R_0}{(\lambda/2)^3} \frac{\omega^2}{\omega^2-\omega_0^2} \sin \omega t \sum_{p=1}^{\infty}\left(x_{n+p} - 2x_n + x_{n-p}\right) - \frac{4P_a}{\rho} k^2 \sin \omega t \, x_n = 0 \qquad (23)$$

The dispersion relation includes now higher-order harmonics through the long-range interactions:

$$\frac{q'(\kappa)}{2}\omega = \frac{q}{2}\omega\left(1 + \frac{2\omega^2}{(\omega^2-\omega_0^2)} \frac{8R_0}{\pi^2\lambda} \sum_{p=1}^{\infty} \sin^2 p \frac{\kappa\lambda}{2}\right) \qquad (24)$$

Long-range interactions broaden the band through the summation in equation (24).
